# A rare diagnosis of primary fibrosarcoma of the thyroid – Case report and mini-review

**DOI:** 10.20945/2359-4292-2023-0467

**Published:** 2024-07-12

**Authors:** Ekin Yiğit Köroğlu, Kübra Turan, Feride Pinar Altay, Fatma Dilek Dellal Kahramanca, Aydan Kiliçarslan, Bilgehan Karadayi, Oya Topaloğlu, Reyhan Ersoy, Bekir Çakir

**Affiliations:** 1 Ankara Bilkent City Hospital Endocrinology and Metabolism Department Ankara Turkey Ankara Bilkent City Hospital, Endocrinology and Metabolism Department, Ankara, Turkey; 2 Ankara Bilkent City Hospital Medical Pathology Department Ankara Turkey Ankara Bilkent City Hospital, Medical Pathology Department, Ankara, Turkey; 3 Ankara Bilkent City Hospital Radiation Oncology Department Ankara Turkey Ankara Bilkent City Hospital, Radiation Oncology Department, Ankara, Turkey; 4 Ankara Yildirim Beyazit University School of Medicine Endocrinology and Metabolism Department Ankara Turkey Ankara Yildirim Beyazit University School of Medicine, Endocrinology and Metabolism Department, Ankara, Turkey

## Abstract

Malignant mesenchymal thyroid tumors are one of the rarest types of thyroid cancer. Clinically, these tumors present as a rapidly growing thyroid mass. Due to their rarity and nonspecific findings, they are not the first conditions that come to mind during differential diagnosis. We report herein the case of an 87-year-old woman presenting with a rapidly growing thyroid mass in whom the differential diagnosis of anaplastic cancer was challenging. Following work up, the patient was diagnosed with primary fibrosarcoma of the thyroid, a rare type of malignant mesenchymal thyroid tumor. Because she declined surgery and her clinical condition was unsuitable for chemotherapy, she was treated with palliative radiotherapy. Primary thyroid fibrosarcoma is a rare cause of thyroid cancer and should be considered in the differential diagnosis of rapidly growing thyroid masses.

## INTRODUCTION

The most common etiologies of rapidly growing thyroid masses are Riedel’s thyroiditis and thyroid malignancies, particularly anaplastic thyroid carcinoma and lymphoproliferative diseases. Malignant mesenchymal thyroid cancer is rare. We report herein the case of a patient with primary fibrosarcoma of the thyroid, one of the rarest types of malignant mesenchymal thyroid tumors. The patient presented with a hard, fixed, and rapidly growing thyroid mass whose differential diagnosis from other diagnoses, particularly anaplastic carcinoma, was clinically and cytologically challenging. Since primary fibrosarcoma of the thyroid is very rare and only a few cases have been reported, literature data on epidemiology are scant. In rapidly growing thyroid masses, cytological and immunohistochemical features of the mass may help establish the differential diagnosis of primary fibrosarcoma. Although surgery is the main line of treatment in the cases reported to date, radiotherapy and chemotherapy have also been administered in selected cases.

## CASE PRESENTATION

An 87-year-old woman presented at our institution’s outpatient clinic with complaints of hoarseness, weight loss, generalized body pain for 2 months, and a palpable neck mass she had noticed 1 week before. At the appointment, she reported having hypothyroidism for 25 years and type 2 diabetes mellitus for 30 years, both of which were controlled with levothyroxine and intensive insulin treatment, respectively. On physical examination, the patient had a hard, fixed, and painless palpable mass on the right side of the neck measuring about 5 x 5 cm in diameter. The mass was visible from a distance and was categorized as Grade 2 according to the World Health Organization (WHO) goiter classification (1).

**Figure 1:**
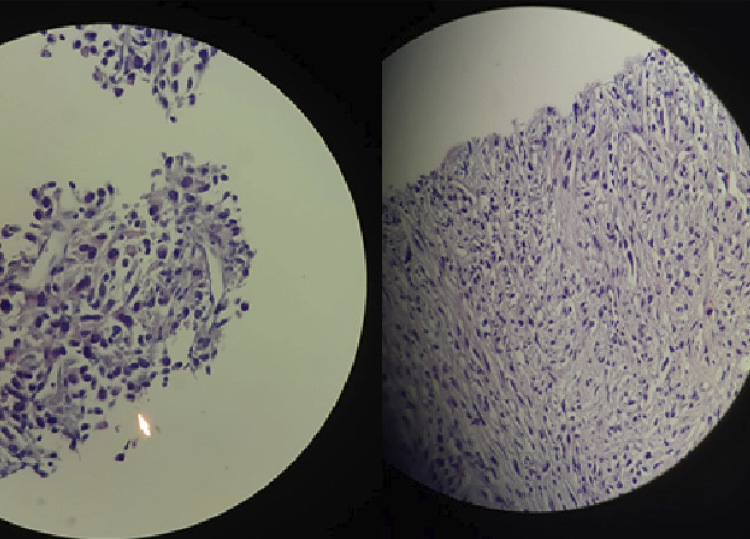
Ultrasound images of the thyroid in the present case. (Left) Nodule with a 2.75 x 5.11 x 6.10 cm in size within the right thyroid lobe. The nodule contained macrocalcifications and areas of cystic degeneration. (Right) Doppler image of the nodule showing no significant blood flow.

On laboratory evaluation, the patient had the following test results (in serum levels, where applicable): thyroid-stimulating hormone (TSH) 1.24 mIU/L (normal range [NR] 0.55-4.78 mIU/L), free thyroxine 1.38 ng/dL (NR 0.89-1.76 ng/dL), free triiodothyronine 3.12 pg/mL (NR 2.3-4.2 pg/mL), antithyroglobulin antibodies > 1,000 IU/mL (NR < 1.3 IU/mL), antithyroid peroxidase antibodies 36 IU/mL (NR < 60 IU/mL), calcitonin < 2 pg/mL (NR < 5 pg/mL), C-reactive protein 224 mg/L (NR 0-5 mg/L), and erythrocyte sedimentation rate 81 mm/hour (NR 0-42 mm/hour). Liver and kidney function tests were within the normal limits. Thyroid ultrasonography revealed a 2.75 x 5.11 x 6.10 cm isoechoic nodule containing macrocalcifications and areas of cystic degeneration within the right lobe, as observed within the limits of measurement (Figure 1). The left lobe had no nodules. The dimensions of the right and left thyroid lobes were, respectively, 3.83 x 5.68 x 8.35 cm and 1.86 x 2.58 x 5 cm.

**Figure 2:**
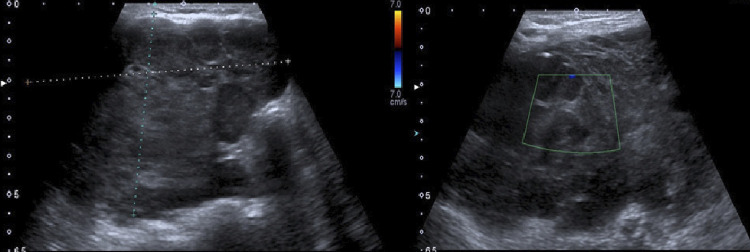
High-power microscopic view of the specimens obtained by fine-needle aspiration biopsy. (Left) Atypical cells in small cohesive groups and a few small micro-tissue fragments with crowded appearance, with rounded/oval dark nuclei (H&E stain, 100x). (Right) Atypical cells with spindle cytoplasm (H&E stain, 100x).

The patient underwent a fine needle aspiration biopsy (FNAB) of the mass. Cytological evaluation revealed abundant groups of degenerated epithelial cells of unidentified origin, along with a few reparative epithelial cells exhibiting atypical features. The final report indicated atypia of undetermined significance. A second FNAB was performed, revealing atypical cells in small cohesive groups and a few small micro-tissue fragments with crowded appearance. These fragments contained cells with rounded/oval dark nuclei, atypical cells with spindle cytoplasm, and isolated spindle cells within fibrin in cell blocks, accompanied by necrosis (Figure 2). The specimen was considered suspicious for malignancy. Lymphoid malignancies were ruled out with cytology and flow cytometric analysis of the FNAB sample. Following these results, the patient was hospitalized with a preliminary diagnosis of anaplastic thyroid carcinoma.

A neck magnetic resonance imaging revealed a 9.0 x 6.5 x 6.5 cm centrally necrotized mass surrounding the right common carotid artery and esophagus. The mass dislocated the esophagus to the left and narrowed the piriform sinus superiorly. On laryngological examination, the right vocal cord was paralyzed in the paramedian position. No significant narrowing of the airway was observed.

An immunocytochemical study of the second FNAB showed the cells stained for vimentin but not for thyroid transcription factor-1 (TTF-1), paired-box gene 8 (PAX8), p40, or thyroglobulin. Following this result, spindle cell mesenchymal tumor was considered to be the most probable diagnosis, and a tru-cut biopsy was performed for a more precise diagnosis. Histopathological evaluation of the specimens revealed neoplastic infiltration consisting of fascicular shapes within a fibrous stroma. A large number of mitotic figures was notable. On immunohistochemistry of the tru-cut specimens, no staining was observed for TTF-1, cluster of differentiation 34 (CD34), PAX8, cytokeratin 7 (CK7), p63, smooth muscle actin (SMA), desmin, S100, synaptophysin, or calcitonin. The Ki-67 proliferation index was 90% and testing for the B-raf proto-oncogene mutation was negative in tumor cells. Considering these morphological and immunohistochemical findings, the diagnosis of primary thyroid fibrosarcoma was considered.

Because no thyrocytes were present in the specimen, 18-F-fluorodeoxyglucose positron emission tomography-computed tomography (18F-FDG PET-CT) scanning was obtained to determine whether the lesion was metastatic or originated primarily from the thyroid. The result revealed a central ametabolic mass lesion (possibly necrosis) measuring 6 x 5.5 x 9 cm, which occupied almost the entire right thyroid lobe (maximum standardized uptake value [SUVmax] of 20.31 g/mL). Multiple millimetric nodules with increased FDG uptake (SUVmax 11.59 g/mL) were observed in both lungs, compatible with metastases.

A multidisciplinary tumor board recommended surgery, which the patient and her relatives declined. Chemotherapy was not considered because of the patient’s general condition and poor performance status (Eastern Cooperative Oncology Group Performance Score [ECOG-PS] 4). Ten sessions of palliative radiotherapy were scheduled after computed tomography simulation. After radiotherapy, the patient presented a partial improvement in pain and compressive symptoms. However, the symptoms other than pain progressed during follow-up and 3 months after radiotherapy the patient died of respiratory failure secondary to aspiration pneumonia.

## DISCUSSION

Primary thyroid sarcomas comprise 0.01%-1.5% of all primary thyroid tumors, with primary thyroid fibrosarcomas making up 9.2% of all thyroid sarcomas (2). This indicates that primary thyroid fibrosarcomas are indeed very rare. Given that this is a rare condition with only a few cases reported in the literature, epidemiological data is insufficient. Primary thyroid fibrosarcomas usually present with a rapidly growing neck mass leading to difficulty swallowing, hoarseness, and/or shortness of breath (3). In most cases, the patients are euthyroid (3-6), although in our case the patient had hypothyroidism. Specific clinical and radiological findings of thyroid fibrosarcomas are unavailable. A definitive diagnosis can be established by pathological and immunohistochemical findings (6). Table 1 summarizes the characteristics of the primary thyroid fibrosarcoma in the present case and in other cases reported in the literature.

**Table 1:** Characteristics of the present case (Case # 1) and other cases of primary thyroid fibrosarcoma described in the literature (Cases # 2-14)

	Age/Sex	Year	Tumor size	Presence of metastasis	Treatment	Thyroid function status
Case # 1	87/F	2022	9.0 x 6.5 x 6.5 cm	Yes (lungs)	RT	Hypothyroidism
Case # 2 (3)	67/M	2016	5.4 x 4.8 cm 6.9 x 5.8 cm (2 masses)	No	Surgery + RT + CT	Euthyroidism
Case # 3 (4)	89/F	2013	10 x 9.5 x 10 cm, 6 x 7 x 13 cm in the right and left lobes, respectively	No	Surgery	Euthyroidism
Case # 4 (5)	54/F	2007	6.1 cm	No	Surgery + RT	Euthyroidism
Case # 5 (6)	81/M	2020	9.5 x 7.2 x 14.5 cm	No	Surgery	Euthyroidism
Case # 6 (9)	59/M	2016	8.2 x 7.6 cm	No	RT + CT	Euthyroidism
Case # 7 (10)	70/M	1996	10 x 12 cm	No	Debulking surgery + RT	Euthyroidism
Case # 8 (11)	74/F	2014	6 cm in the long axis	Yes (cervical lymph nodes)	Surgery + CT	Euthyroidism
Case # 9 (12)	69/M	1976	14 x 8 x 7 cm	No	Surgery	Euthyroidism
Case # 10 (13)	31/M	2007	7 x 5 cm	No	Surgery + RT	Euthyroidism
Case # 11 (14)	71/M	2014	8 x 9 cm	No	Surgery + RT	Euthyroidism
Case # 12 (15)	76/F	1957	7 x 4 cm	No	RAI + Surgery	Euthyroidism
Case # 13 (16)	38/F	1946	N/A	N/A	RT	Hyperthyroidism
Case # 14 (17)	32/F	1954	6 x 3.5 cm	Yes (cervical lymph nodes)	Surgery	Euthyroidism

Abbreviations: CT, chemotherapy; F, female; M, male; N/A, not available; RT, radiotherapy; RAI, radioactive iodine.

The present case involved two stages in the process of cytological diagnosis. In the first stage, the differential diagnosis of the rapidly growing thyroid mass was considered after FNAB, and a preliminary diagnosis of malignant mesenchymal tumor was established. In the second stage, by considering cytological and immunohistochemical differences between mesenchymal tumor subtypes, a final diagnosis was reached.

The differential diagnosis of rapidly growing thyroid mass includes Riedel’s thyroiditis, fibrous variant of Hashimoto’s thyroiditis, medullary thyroid carcinoma, anaplastic thyroid carcinoma, squamous cell cancer, diffuse sclerosing papillary thyroid carcinoma, sarcoma, hemangiopericytoma, paraganglioma, lymphoma, and nerve sheath tumors (*e.g.*, schwannoma) (7). In the present case, no thyrocytes were present in the FNAB specimens, rendering less likely the diagnoses of Riedel’s thyroiditis, papillary thyroid carcinoma, and Hashimoto’s thyroiditis. The absence of characteristic findings or immunochemical staining for calcitonin, p40, cytokeratin, and neuron-specific markers excluded the diagnoses of medullary thyroid carcinoma, squamous cell cancer, anaplastic thyroid carcinoma, hemangiopericytoma, and paraganglioma, respectively. The most challenging preliminary diagnosis in the differential diagnosis was anaplastic thyroid carcinoma, particularly two of its subtypes, as they may be confused with malignant mesenchymal tumors. The spindle cell variant of anaplastic carcinoma may resemble mesenchymal cancers morphologically. In this variant, the appearance of epithelial structures (staghorn branching or hemangiopericytoma-like vascularity) and staining for epithelial markers (*e.g.*, keratin) suggest the diagnosis of anaplastic thyroid carcinoma. Notably, rhabdomyosarcomatous differentiation of anaplastic thyroid carcinoma is characterized by weak staining for epithelial markers and strong staining for vimentin, actin, desmin, and myoglobin (7). In the present case, there were no thyrocytes or staining for epithelial markers in the cytological specimens. Additionally, the staining for vimentin only and the absence of staghorn branching or hemangiopericytoma-like vascularity in the samples excluded the diagnosis of anaplastic carcinoma. Hemangiopericytoma was also excluded because of the absence of the typical uniform “staghorn” vascular pattern in this type of tumor. The absence of peripheral nerve elements ruled out the possibility of nerve sheath tumors. The diagnosis of lymphoma was ruled out by flow cytometry. Finally, the vimentin staining and the microscopic appearance of malignant cells led to the diagnosis of malignant mesenchymal tumor.

**Figure 3:**
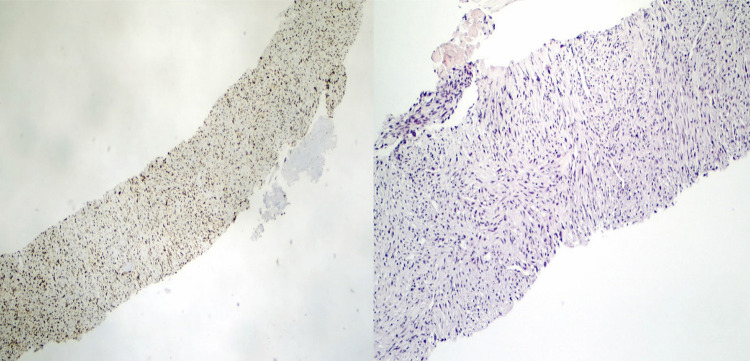
Low-power microscopic view of the specimens obtained by tru-cut needle biopsy showing atypical spindle cells arranged in a herringbone pattern, indicative of fibrosarcoma (H&E stain, 40x).

A review by Gubbi and cols. reported 530 cases of thyroid mesenchymal tumors. Malignant mesenchymal tumor subtypes include malignant nerve sheath tumors, malignant vascular tumors, rhabdomyosarcoma, leiomyosarcoma, liposarcoma, osteosarcoma, Ewing sarcoma, synovial sarcoma, fibrosarcoma, chondrosarcoma, pleomorphic sarcoma, and carcinosarcoma. Cytological features and immunohistochemical staining are essential in differentiating these subtypes. Staining for S-100 can be seen on peripheral nerve sheath tumors, liposarcomas, and chondrosarcomas. Malignant vascular tumors are characterized by CD34 staining. Staining with desmin can be detected in rhabdomyosarcoma or leiomyosarcoma, while CD99, p40, or pankeratin staining is seen in Ewing sarcoma (8). In the present case, the staining results were negative for all these markers, except for vimentin, which was the only marker to show positive staining. Staining for vimentin has been reported in osteosarcomas, fibrosarcomas, and carcinosarcomas (8). The absence of both epithelial components and osteoblast-like cells with pleomorphic nuclei and osseous metaplasia in the cytological material excluded the diagnoses of carcinosarcoma and osteosarcoma, respectively (8). The identification of spindle cells arranged in a herringbone pattern in the smears was indicative of fibrosarcoma (Figure 3). Considering these findings, the diagnosis of primary fibrosarcoma of the thyroid was established.

Surgery is the primary treatment for patients with thyroid fibrosarcoma. If the mass cannot be completely removed during surgery, adjuvant radiotherapy may be given. In high-grade fibrosarcomas, chemotherapy is also an option. Apart from traditional chemotherapy directed against sarcomas, targeted therapies have also been considered as a treatment alternative. Clinical studies on the effectiveness of mammalian target of rapamycin (mTOR) inhibitors and tyrosine kinase inhibitors are ongoing (5). Chemotherapy and radiotherapy can be used as alternatives in patients who are unsuitable for surgery (8). Because the patient in the present case declined surgery and her medical condition was unsuitable both for surgery and chemotherapy, she was treated with radiotherapy. The patient was then followed up for 3 months and the severity of her symptoms decreased in the first month. After that, dyspnea, dysphagia, and hoarseness progressed, and the patient died 3 months after radiotherapy due to respiratory failure secondary to aspiration pneumonia.

Among the cases shown in Table 1, ours was the only one with distant metastasis. This suggests that fibrosarcoma not only progresses rapidly and aggressively in the thyroid but may be prone to metastasis, as in fibrosarcomas that develop in other organs. Of all reported cases, ours was the only one with hypothyroidism.

In conclusion, primary thyroid fibrosarcoma is a rare cause of thyroid cancer that should be considered in the differential diagnosis of rapidly growing thyroid masses. For a faster diagnosis in rapidly growing thyroid masses, tru-cut biopsy or excisional biopsy may be considered preferentially instead of FNAB, as detailed immunohistochemical studies are helpful in establishing the differential diagnosis.
